# Mbnl1-mediated alternative splicing of circMlxipl regulates Rbbp6-involved ChREBP turnover to inhibit lipotoxicity-induced β-cell damage

**DOI:** 10.1186/s10020-024-00991-9

**Published:** 2024-11-23

**Authors:** Yingying Gong, Meilin Wei, Xiaopei Cao, Changliu Xu, Jiewen Jin, Ling Pei, Yanbing Li, Haipeng Xiao, Liting Wu

**Affiliations:** 1 Department of Geriatrics, The First Affiliated Hospital, Sun Yat-Sen University, Guangzhou, 510080 China; 2 Department of Endocrinology and Metabolism, The Second Affiliated Hospital of Nanchang University, Nanchang, 330006 China; 3 Department of Endocrinology, The First Affiliated Hospital, Sun Yat-Sen University, Guangzhou, 510080 China; 4 Department of Endocrinology and Metabolism, First Affiliated Hospital of Jinan University, Guangzhou, 510632 China

**Keywords:** circMlxipl, Mbnl1, Rbbp6, Hdac3, ChREBP, Lipotoxic β-cells

## Abstract

**Background:**

Diabetes, a global epidemic, is the leading cause of mortality globally. The aim of this study is to get better understanding of pathophysiology of diabetes.

**Methods:**

Palmitic acid (PA)-treated β-cells, db/db mice and high fat diet (HFD)-fed mouse model of type 2 diabetes were established. H&E was used to assess the histological changes of pancreas. IHC, FISH, western blot or qRT-PCR was employed to detect the expression of key molecules in primary islets or lipotoxic β-cells. Cell behaviors were detected by MTT, EdU incorporation assay, TUNEL assay and glucose-induced insulin secretion (GSIS). The associations among circMlxipl, Mbnl1 and Rbbp6 were validated by RIP and RNA pull-down assays, and the direct binding between Hdac3 and Mbnl1 promoter was examined by ChIP and luciferase assays. Co-IP was employed to assess the interaction between ChREBP and Rbbp6, as well as the ubiquitination of ChREBP.

**Results:**

Hdac3 and ChREBP were upregulated, but Mbnl1 and circMlxipl were downregulated in islets from diabetic mice and lipotoxic β-cells. Mbnl1 overexpression protected against PA-induced impairments in lipotoxic β-cells through modulating back-splicing of circMlxipl and suppressing ChREBP. Hdac3 served as a transcriptional repressor of Mbnl1, and it was implicated in circMlxipl-mediated protection via regulating ChREBP expression in lipotoxic β-cells. Lack of circMlxipl inhibited Rbbp6-mediated ubiquitin-proteasomal degradation of ChREBP in lipotoxic β-cells. In vivo studies revealed that Hdac3 knockdown or Mbnl1 overexpression alleviated diabetes symptoms through circMlxipl-regulated ChREBP in diabetic mice.

**Conclusion:**

Mbnl1-mediated alternative splicing of circMlxipl regulates Rbbp6-involved ChREBP turnover to inhibit lipotoxicity-induced β-cell damage.

## Introduction

Type 2 diabetes mellitus (T2DM), which accounts for > 90% of all diabetes, remains one of the leading causes of death globally (Schleicher et al. 2022; Dludla et al. 2023). It is well-established that T2DM patients are characterized with hyperglycemia resulting from insufficient insulin sensitivity and defective insulin secretion (Galicia-Garcia et al. 2020). Currently, healthy lifestyle interventions and anti-glycemic agents which aim to control plasma glucose level are involved in the treatment of T2DM (Nauck et al. 2021). Unfortunately, the available diabetes treatments have adverse effects, and individualized therapy is required for each patient (Diabetes Canada Clinical Practice Guidelines Expert C 2020). It is urgent to delineate the mechanism underlying the pathophysiology of diabetes and develop novel targeted therapy for diabetes. Pancreatic β-cells contribute to insulin secretion, and defects in β-cell function and mass play crucial roles in T2DM development and progression (Dludla et al. 2023; Galicia-Garcia et al. 2020). Previous studies have illustrated that ChREBP, also known as Mlxipl, is upregulated in diabetes and responsible for β-cell apoptosis and loss of β-cell mass (Jing et al. 2016; Katz et al. 2022). However, the regulatory mechanism of ChREBP-mediated β-cell apoptosis remains ambiguous.

circular RNAs (circRNAs), a class of closed non-coding RNAs, are generated by back-splicing (Huang et al. 2020). Bioinformatics analysis based on CIRCpedia database (https://www.picb.ac.cn/rnomics/circpedia/search) predicted that circMlxipl was formed from back-splicing Mlxipl exons. More importantly, circMlxipl is downregulated in diabetes, and subsequent experiments support that circMlxipl is regulated by non-esterified fatty acid (NEFA) (Wu et al. 2020). The role of circMlxipl in diabetes remains elusive, and if circMlxipl regulates β-cell failure by modulating ChREBP expression merits further investigation.

RBPDB database (http://rbpdb.ccbr.utoronto.ca/advanced_search.php) predicted seven putative RNA binding proteins (RBPs, including Fus, Srsf1, Mbnl1, Srsf13a, Rnmy1a1, Pabpc, Eif4b) of Mlxipl 5’ splice site of exon 3 and 3’ splice site of exon 7. Among these candidates, muscleblind-like 1 (Mbnl1) attracted our attentions due to its significant reduction in response to palmitic acid (PA) treatment. Moreover, Mbnl1 facilitates alternative splicing of exon 11 in *INSR* (insulin receptor) through direct interaction with intronic enhancer (Sen et al. 2010). Mbnl1 is induced by insulin and decreased in β-cells upon high glucose treatment (Malakar et al. 2016). Furthermore, our preliminary data also revealed that Mbnl1 was downregulated in diabetic mouse model and lipotoxic β-cells, suggesting that Mbnl1 might act as an RBP of pre-Mlxipl to regulate β-cell failure in diabetes. A type I histone deacetylase (Hdac), namely Hdac3, was predicted as a transcription factor of Mbnl1 by AnimalTFDB. Intriguingly, the selective Hdac3 inhibitors protects against cytokine- or palmitate-induced β-cell apoptosis (Chou et al. 2012; Meier and Wagner 2014; Plaisance et al. 2014). Recently, it has been reported that inhibition of Hdac3 ameliorates T2DM-induced endothelial dysfunction through modulating Nrf2 (Huang et al. 2021). The protective role of Hdac3 inhibitor raises the possibility that Hdac3 might contribute to the transcriptional regulation of Mbnl1, thereby modulating the back-splicing of circMlxipl and β-cell failure.

Ubiquitin–proteasome pathway is a classic protein degradation pathway, and E3 ubiquitin ligase specifically recognizes the substrate and catalyzes its ubiquitination (Gupta et al. 2021). Rbbp6 was predicted as an E3 ubiquitin ligase of ChREBP by ubibrowser (http://ubibrowser.bio-it.cn/ubibrowser_v3/), and there was a putative association between Rbbp6 and circMlxipl. Our preliminary data showed that circMlxipl and Rbbp6 co-localized to the cytosol of β-cells, suggesting that the interaction between circMlxipl and Rbbp6 might contribute to Rbbp6-mediated ChREBP turnover.

We thus hypothesized that upregulated Hdac3 transcriptionally suppressed Mbnl1 expression, thus inhibiting the back-splicing of circMlxipl in lipotoxic β-cells. The impaired association between circMlxipl and Rbbp6 inhibited Rbpp6-mediated degradation of ChREBP, thereby triggering β-cell apoptosis. Our findings put a greater spotlight on the underlying mechanism of β-cell faliure, and circMlxipl and its associated molecules have been identfied as promising therapeutic targets for diabetes.

## Materials and methods

### Animal study

All animal studies were approved by the committee for Clinical Research and Animal Trials of the First Affiliated Hospital of Sun Yat-sen University (Research Protocol [2017]056). Male db/db mice (5-week-old, n = 8 per group), age-matched wild-type littermate db/m mice (n = 8/group) and male C57BL/6 J mice (5-week-old, n = 8/group) were obtained from Hunan Slack Jingda experimental animal (Hunan, China). All mice were housed at room temperature with 12 h/12 h light/dark cycle and free access to water and chow food. C57BL/6 J mice were fed high fat diet (HFD, D12492, Research Diets, NJ, USA) or normal control diet (NFD, D12450J, Research Diets) for 8 months as previous described (Wu et al. 2020). Adeno-associated viral (AAV) constructs driven by the mouse insulin promoter, including rAAV8-sh-NC, rAAV8-sh-Hdac3, rAAV8-ov-NC and rAAV8-ov-Mbnl1, and AAV helper vector were from Fenghbio (Changsha, Hunan, China). For in vivo functional studies, mice were randomly divided into six groups (n = 8/group): NFD, HFD, HFD + rAAV8-sh-NC, HFD + rAAV8-sh-Hdac3, HFD + rAAV8-ov-NC and HFD + rAAV8-ov-Mbnl1. C57BL/6 J mice were administrated with AAV vector (1 × 10^11^ viral genome (vg)/mouse in 100 μL) into the pancreatic duct 2 months before subsequent analysis.

### Glucose tolerance tests (GTTs) and insulin tolerance tests (ITTs)

Blood glucose was measured from tail vein using a Glucometer Elite monitor (Abbott, Oxon, UK). GTTs were performed by intraperitoneal (i.p.) injection of D-glucose (1 g/kg) after overnight fasting. ITTs were performed by i.p. injection of 1.2 U/kg insulin after 5 h of fasting. Mice blood samples were collected and were centrifuged at 3,000 g for 15 min to collect serum. The insulin level was measured using Rat/Mouse Insulin ELISA (EZRMI-13 K, Millipore, Billerica, MA, USA).

### Hematoxylin and Eosin (H&E) staining

Paraffin-embedded sections were rehydrated and stained with H&E (Sigma-Aldrich, St. Louis, MO, USA). Images were photographed under a microscope (Nikon, Tokyo, Japan).

### Primary islet isolation and culture

Mouse islets were isolated as previous described (Corbin et al. 2021). Briefly, pancreas was dissected and digested using collagenase (Gibco, Grand Island, NY, USA). The digestion was terminated with KRBH balanced buffer with 0.1% BSA and 2.5 mM glucose. Islets were separated by density gradient and cultured in RPMI1640 containing 10% FBS (Gibco) at 37 °C/5% CO_2_.

### Cell culture, transfection and treatment

Mouse pancreatic β-cell line Min6 cells were from ATCC (Manassas, VA, USA), and grown in DMEM containing 15% FBS (Gibco), 50 μM β-mercaptoethanol and 25 mM Glucose. Min6 cells were treated with 0.5 mM palmitate acid (PA, Sigma-Aldrich) for 1 day, and 0.1% BSA was used as a vehicle control. sh-circMlxipl, sh-Hdac3 and sh-Rbbp6 were obtained from Fenghbio (Changsha, Hunan). circMlxipl was cloned into pCDH vector (Youbio, Changsha), and Mbnl1 cDNA was cloned into pcDNA3.1 (Youbio). Min6 cells were transfected with shRNA or/and overexpression plasmid using Lipofectamine 3000 (Invitrogen). Min6 cells were treated with protein synthesis inhibitor 5 μg/mL cycloheximide (CHX, Sigma-Aldrich) for 0, 15, 30, 60, 120 or 240 min. To block ubiquitin-proteasomal pathway, Min6 cells were incubated with MG132 (20 µM, Sigma-Aldrich) for 12 h.

### qRT-PCR

Total RNA was extracted from mouse islets or Min6 cells using Trizol (Invitrogen). After reverse transcription using SuperScript III (Invitrogen), cDNA was amplified using SYBR Green MasterMix (ABI, Foster City, CA, USA) with ABI 7900 Real-Time PCR (ABI). Gene expression was calculated using 2^−ΔΔCT^ method with GAPDH or U6 used as an internal control.

### RNase R/Actinomycin D resistant assays

2 μg RNA was incubated with RNase R (3 U/μg, Epicenter Technologies, Madison, WI, USA) for 0, 10, 20, 30 and 40 min at 37 °C. For Actinomycin D treatment, Min6 cells were treated with actinomycin D (5 μg/mL, Sigma-Aldrich) for 0, 12 and 24 h. The levels of circMlxipl and Mlxipl mRNA were detected by qRT-PCR. circMlxipl forward primer, 5’- CCAGAAGATGCTTATGTTGG-3’, reverse primer, 5’-TCCAGGATGACAGCCTCAGG-3’; Mlxipl mRNA forward primer, 5’- *CGTAAGTCCAGCAGGGAAGG-3’; reverse primer, 5’- GGTGAAGAGTGTGTCGGAGA-3’.*

### Immunohistochemistry (IHC) analysis

Paraffin-embedded mouse pancreas sections were then deparaffined and subjected to antigen retravel, followed by the blocking with 1% BSA. The slides were incubated with primary antibody at 4 °C overnight, and secondary antibody-HRP (Invitrogen). Signals were detected using DAB substrate (Beyotime). Primary antibodies: anti-Ki67 (1:100, ab15580, Abcam, Cambridge, UK), anti-Hdac3 (1:100, ab137704, Abcam), anti-Mbnl1 (1:200, PA5-143882, Invitrogen) and anti-ChREBP (1:100, ab92809, Abcam) antibodies.

### MTT assay

Min6 cells (1 × 10^4^ per well) were seeded onto 96-well plates 24 h prior to BSA or PA treatment. At 24 h post-treatment, MTT solution (10 μL/well, Beyotime) was incubated with Min6 cells for 4 h, followed by the incubation with Formazan (100 μL/well). A570 was assessed using a BioTek microplate reader (Winooski, VT, USA). Cell viability was presented as a percentage of the BSA control.

### EdU incorporation assay

EdU incorporation was examined using Cell-Light EdU Kit (ThermoFisher scientific). Briefly, Min6 cells were treated with BSA or PA for 24 h, and incubated with 10 μM EdU for 4 h. Cells were then fixed with 4% PFA and incubated with Apollo mixture for 1 h. Nucleus was detected by DAPI. EdU-positive cells were photographed and counted under a confocal microscope (Nikon).

### TUNEL assay

Cell apoptosis was examined using Click-iT Plus TUNEL Assay kit (Invitrogen). In brief, fixed and permeabilized cells were incubated with TdT reaction buffer, followed by the incubation with TdT reaction cocktail for 1 h at 37 °C. Click-iT reaction was performed at room temperature for 30 min. Nucleus was visualized by Hoechst 33342. TUNEL-positive cells were photographed and counted under a confocal microscope (Nikon).

### Glucose-induced insulin secretion (GSIS)

GSIS was conducted as previous described (Robson-Doucette et al. 2011). In brief, Min6 cells were pre-treated with 2.8 mM glucose Krebs–Ringer HEPES (KRH) buffer for 1 h, followed by the incubation with 16.7 mM glucose RPMI1640. The insulin level was measured using Rat/Mouse Insulin ELISA (EZRMI-13 K, Millipore, Billerica, MA, USA).

### Western blot

Total protein lysates were prepared using RIPA lysis buffer (Beyotime). Nuclear and cytoplasmic extracts were lysed using NE-PER Reagents (Pierce). Equal amounts of proteins were separated by SDS-PAGE, and transferred onto PVDF membrane (Beyotime). The blots were probed with primary antibody at 4 °C overnight. After blocking, and HRP-conjugated secondary antibody (Invitrogen). The bands were visualized using SuperSignal West Pico PLUSE ECL substrate (Pierce). Primary antibodies used in western blot: anti-Hdac3 (1:2000, ab137704, Abcam), anti-Mbnl1 (1:1000, PA5-143882, Invitrogen), anti-ChREBP (1:1000, ab92809, Abcam), anti-Fus (1:1000, ab243880, Abcam), anti-Srsf1 (1:250, 32-4500, Invitrogen), anti-Rbbp6 (1:1000, PA5-23054, Invitrogen), anti-Gapdh (1:2000, ab8245, Abcam), anti-Lamin B1 (1:1000, ab256380, Abcam) and anti-β-actin (1:3000, ab8226, Abcam) antibodies.

### RNA fluorescence in situ hybridization (FISH)

Cy3-labeled circMlxipl probe (5’-Cy3-AAGAGCTGTTCGCACCATCGCTCTGGTGGCG-3’) was synthesized by GenePharma (Shanghai, China). Min6 cells were fixed with 4% PFA, and RNA FISH was conducted using Fluorescent in situ Hybridization Kit (GenePharma). Nucleus was detected by DAPI. Images were photographed under a confocal microscope (Nikon).

### RNA pull-down assay

RNA pull-down assay was conducted using Pierce RNA Pull-Down Kit (20164, Pierce). In brief, pre-Mlxipl or circMlxipl probe was labeled with desthiobiotin, and conjugated to streptavidin beads. Cell lysates were then incubated with the beads at 4 °C overnight, and the elutes were analyzed by western blot. Antisense probe or whole cell lysates acted as a negative or an input control, respectively.

### RNA immunoprecipitation (RIP) assay

RIP assay was conducted using Magna RIP Kit (17-700, Millipore). Briefly, Min6 cells were lysed using RIP Lysis Buffer. Anti-Mbnl1 (2 μg, ab309348, Abcam) antibody -conjugated beads were incubated with cell lysates at 4 °C overnight. The enriched pre-Mlxipl was analyzed by qRT-PCR.

### Chromatin immunoprecipitation (ChIP) assay

ChIP assay was conducted using Pierce Agarose ChIP Kit (26156, Pierce). Briefly, Min6 cells were treated with 1% formaldehyde, and the chromatin fractions were prepared by MNase digestion. The chromatins were then incubated with anti-Hdac3 (2 μg, ab137704, Abcam) antibody or normal IgG at 4 °C overnight. Purified DNA was analyzed by qRT-PCR.

### Dual-luciferase assay

The wild-type (WT) Mbnl1 promoter region (−2000/ + 100 bp) containing BS1 (-324/-318) and BS2 (-882/-876), or the mutants (MUT) of BS1 and/or BS2 were cloned into pGL-3 (Promega, Madison, WI, USA). Min6 cells were co-transfected with Hdac3 overexpression construct and WT, MUT1 (BS1 mutant), MUT2 (BS2 mutant) or MUT1&2 (double mutant of BS1 and BS2). For knockdown study, Min6 cells were co-transfected with sh-NC/sh-Hdac3 and WT/MUT Mbnl1, followed by PA treatment. At 48 h post-transfection, the luciferase activity was examined using Dual Luciferase Reporter System (Promega).

### co-immunoprecipitation (co-IP)

Min6 cells were lysed with IP lysis buffer (Beyotime). Cell lysates were incubated with anti-Rbbp6 (2 μg, PA5-23054, Invitrogen), anti-ChREBP (2 μg, PA1-16806, Invitrogen) or normal rabbit IgG. The antigen–antibody complexes were then enriched by Protein A beads (Pierce), and the elutes were analyzed by western blot.

### Statistical analysis

All experiments were conducted at least 3 times, and data were presented as means ± S.D. Two-group comparison was analyzed by Student’s *t* test, and multi-group comparison was assessed by one-way ANOVA followed by Tukey's post hoc test or two-way repeated-measures ANOVA followed by Bonferroni's post hoc test using the SPSS22.0. *P* < 0.05 was considered to be statistically significant.

## Results

### Hdac3 and ChREBP are upregulated, but Mbnl1 and circMlxipl are downregulated in islets from diabetic mice and lipotoxic β-cells

**Fig. 1:**
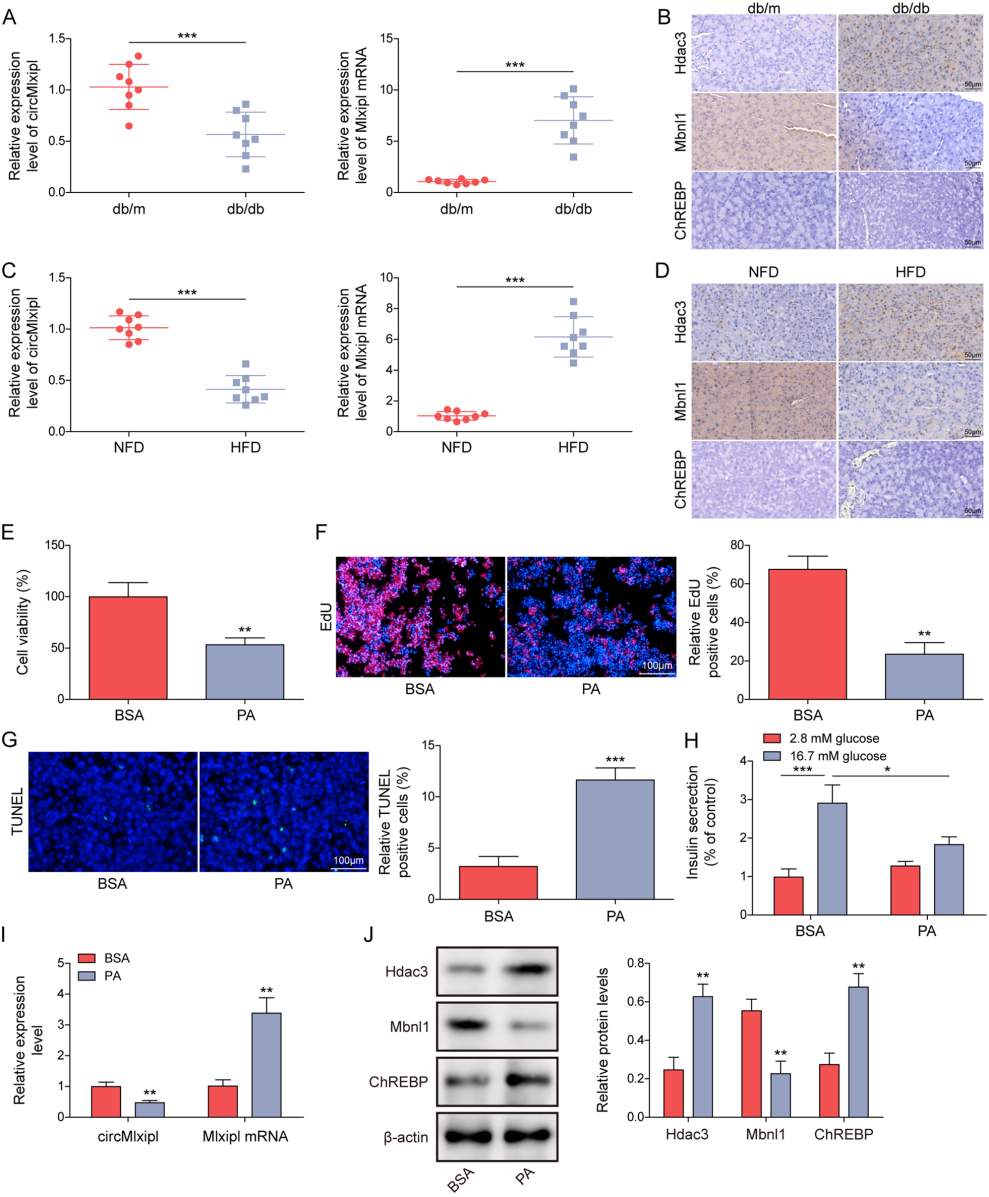
Hdac3 and ChREBP are upregulated, but Mbnl1 and circMlxipl are downregulated in islets from diabetic mice and lipotoxic β-cells. **A**, **C** The levels of circMlxipl and Mlxipl mRNA in mouse islets were detected by qRT-PCR. **B**, **D** The immunoreactivities of Hdac3, Mbnl1 and ChREBP in mouse islets were detected by IHC. Scale bar, 50 μm. **E** Cell viability of Min6 cells was monitored by MTT assay. **F** Cell proliferation was detected by EdU incorporation assay with quantitative analysis. Scale bar, 100 μm. **G** Cell apoptosis was assessed by TUNEL assay with quantitative analysis. Scale bar, 100 μm. **H** Insulin secretion was detected by GSIS. **I** The levels of circMlxipl and Mlxipl mRNA in Min6 cells were detected by qRT-PCR. **J** The protein levels of Hdac3, Mbnl1 and ChREBP in Min6 cells were detected by western blot. *P < 0.05; **P < 0.01; ***P < 0.001

To test the hypothesis, we first examined the expression pattern of key molecules. qRT-PCR showed that circMlxipl was remarkably decreased in primary islets from db/db mice and HFD C57BL/6 J mice, compared with corresponding controls. By contrast, Mlxipl mRNA exhibited an opposite trend (Fig. 1A, C). The immunoreactivities of Hdac3 and ChREBP were markedly increased in primary islets from db/db mice and HFD mice, while the expression of Mbnl1 was decreased in diabetic islets as detected by IHC analysis (Fig. 1B, D). An in vitro model of PA-induced lipotoxicity was next established in Min6 cells. MTT and EdU incorporation assays showed that PA impaired the viability and proliferation of Min6 cells (Fig. 1E, F). The apoptotic rate of PA-treated Min6 cells was dramatically increased as detected by TUNEL assay (Fig. 1G), and this was accompanied with the decrease of insulin secretion (Fig. 1H). Consistent with the in vivo findings, circMlxipl was greatly downregulated in PA-treated Min6 cells, while PA induced Mlxipl mRNA level in Min6 cells (Fig. 1I). In addition, western blot revealed that Hdac3 and ChREBP were upregulated by PA, but Mbnl1 was downregulated by PA in Min6 cells (Fig. 1J). These data suggest that Hdac3 and ChREBP are increased, but Mbnl1 and circMlxipl are decreased in islets from diabetic models and lipotoxic β-cells.

### Overexpression of circMlxipl rescues PA-induced impairments in lipotoxic β-cells through decreasing ChREBP

**Fig. 2:**
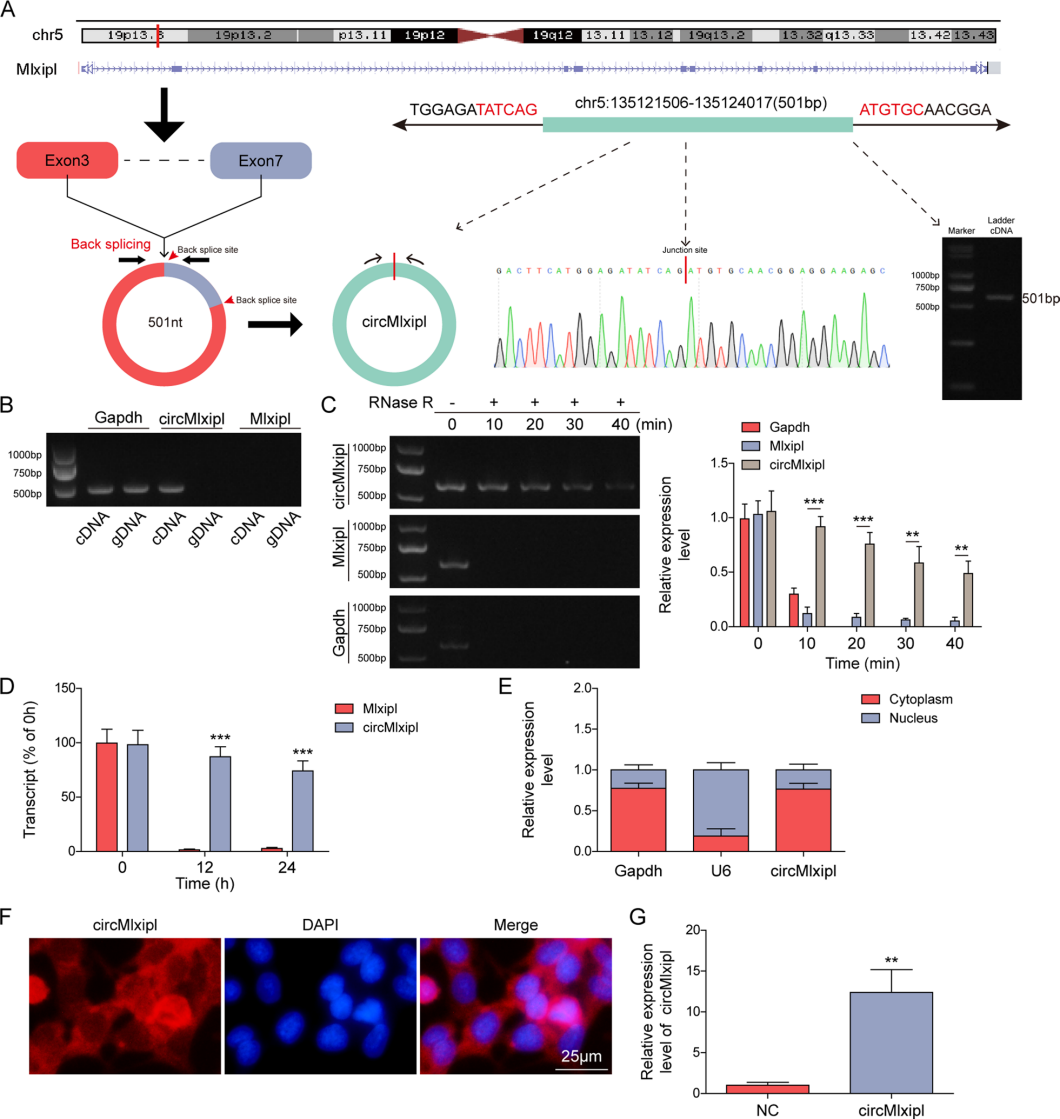

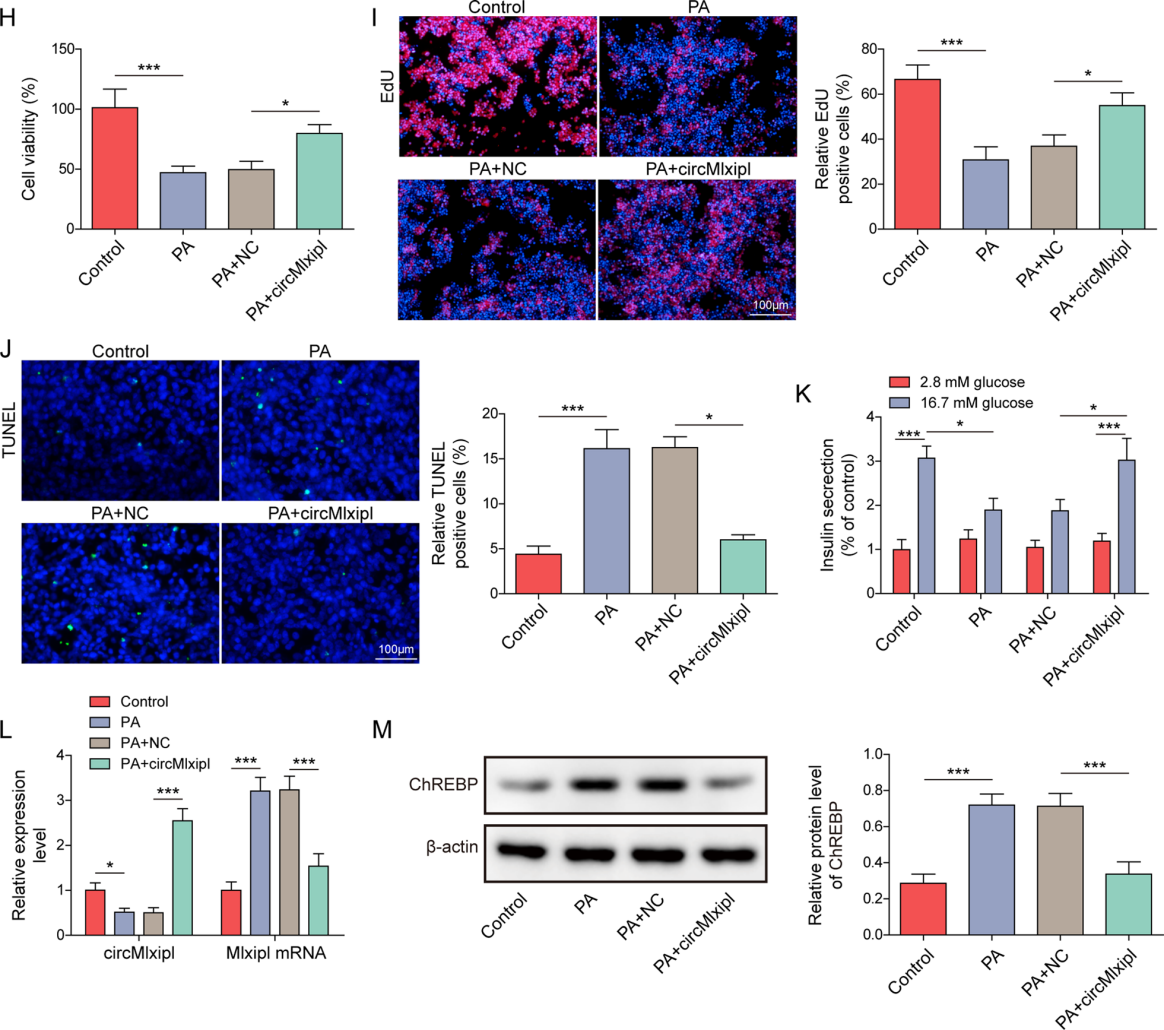
Overexpression of circMlxipl rescues PA-induced impairments in lipotoxic β-cells through decreasing ChREBP. **A** circMlxipl was formed by back-splicing, and the sequence of circMlxipl was confirmed by Sanger sequencing and gel electrophoresis. **B** The validation of back-spliced junction sites by divergent primers. **C**, **D** The stabilities of circMlxipl and Mlxipl mRNA were assessed by RNase R/Actinomycin D resistance assays. **E** The nuclear and cytoplasmic expression of circMlxipl were detected by subcellular fractionation and qRT-PCR. **F** The subcellular localization of circMlxipl was detected by RNA FISH. Scale bar, 20 μm. **H** Cell viability of Min6 cells was monitored by MTT assay. **I** Cell proliferation was detected by EdU incorporation assay with quantitative analysis. Scale bar, 100 μm. **J** Cell apoptosis was assessed by TUNEL assay with quantitative analysis. Scale bar, 100 μm. **K** Insulin secretion was detected by GSIS. **L** The levels of circMlxipl and Mlxipl mRNA in Min6 cells were detected by qRT-PCR. **M** The protein level of ChREBP in Min6 cells was detected by western blot. *P < 0.05; **P < 0.01; ***P < 0.001

circMlxipl (501 bp in length) was generated when the 5’ splice site of exon 3 was joined to the 3’ splice site of exon 7, and the sequence of circMlxipl was confirmed by Sanger sequencing (Fig. 2A). In addition, the back-splicing fragment of circMlxipl was successfully amplified by the divergent primers from cDNA, but not from gDNA. By contrast, the divergent primers failed to amplify Mlxipl mRNA from neither cDNA nor gDNA (Fig. 2B). RNase R/actinomycin D resistance assays further revealed that circMlxipl was more stable than Mlxipl mRNA and GAPDH (Fig. 2C, D). Subcellular fractionation and RNA FISH revealed that circMlxipl was predominantly expressed in the cytoplasm of Min6 cells (Fig. 2E, F). As anticipated, qRT-PCR showed that transfection of circMlxipl overexpression plasmid dramatically induced circMlxipl level in Min6 cells (Fig. 2G). circMlxipl overexpression reversed PA-impaired cell viability and proliferation (Fig. 2H, I). Moreover, PA-triggered apoptosis of Min6 cells was also rescued by circMlxipl overexpression as detected by TUNEL assay (Fig. 2J), along with increased insulin secretion (Fig. 2K). Furthermore, qRT-PCR and western blot revealed that PA-downregulated circMlxipl, as well as PA-upregulated Mlxipl mRNA and ChREBP, were counteracted by circMlxipl overexpression (Fig. 2L, M). Collectively, these findings indicate that circMlxipl exerts protective effects in lipotoxic β-cells, possibly via decreasing ChREBP.

### Mbnl1 overexpression protects against PA-induced impairments in lipotoxic β-cells through modulating circMlxipl/ChREBP

**Fig. 3:**
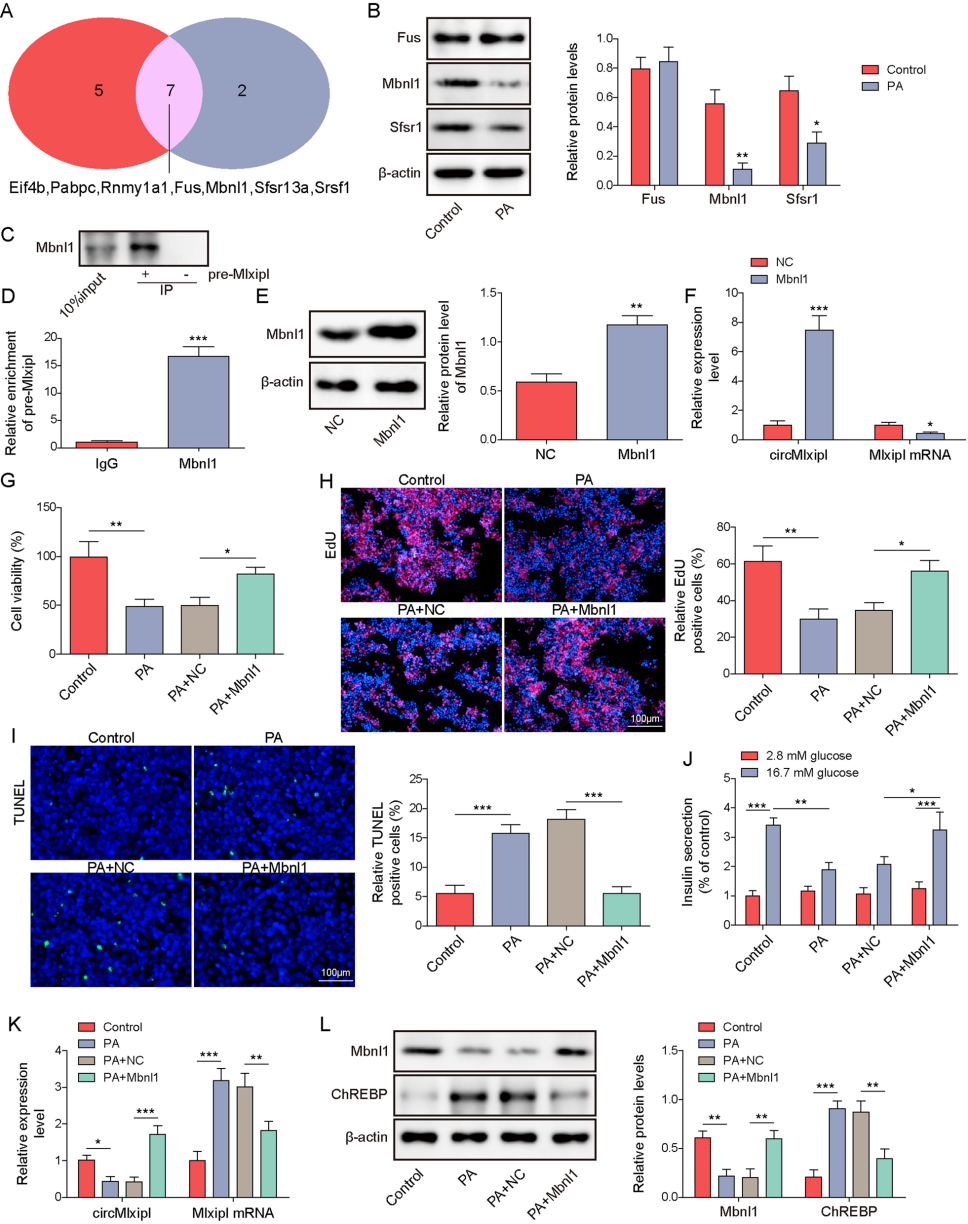
Mbnl1 overexpression protects against PA-induced impairments in lipotoxic β-cells through modulating circMlxipl/ChREBP. **A** The putative RPBs of circMlxipl were predited using RBPDB database. **B** The protein levels of Fus, Mbnl1 and SRSF1 in Min6 cells were detected by western blot. The association between pre-Mlxipl and Mbnl1 was detected by RNA pull-down (**C**) and RIP assays (**D**). Anti-sense probe and normal IgG served as negative controls for RNA pull-down assay and RIP, respectively. **E** The protein levels of Mbnl1 in Min6 cells were detected by western blot. **F** The levels of circMlxipl and Mlxipl mRNA in Min6 cells were detected by qRT-PCR. **G** Cell viability of Min6 cells was monitored by MTT assay. **H** Cell proliferation was detected by EdU incorporation assay with quantitative analysis. Scale bar, 100 μm. **I** Cell apoptosis was assessed by TUNEL assay with quantitative analysis. Scale bar, 100 μm. **J** Insulin secretion was detected by GSIS. **K** The levels of circMlxipl and Mlxipl mRNA in Min6 cells were detected by qRT-PCR. **L** The protein levels of Mbnl1 and ChREBP in Min6 cells were detected by western blot. *P < 0.05; **P < 0.01; ***P < 0.001

Bioinformatics analysis based on RBPDB database predicted 7 putative RBPs, including Eif4b, Pabpc, Rbmy1a1, Fus, Mbnl1, Sfsr13a and Srsf1 (Fig. 3A). Among the three β-cell dysfunction-related RBPs, western blot revealed that PA reduced Mbnl1 and Srsf1 protein levels in Min6 cells. Mbnl1 with more significant reduction was selected for the subsequent investigation (Fig. 3B). RNA pull-down and RIP assays confirmed that there was a direct association between Mbnl1 and pre-Mlxipl (Fig. 3C, D). Additionally, overexpression of Mbnl1 led to the induction of Mbnl1 and circMlxipl in Min6 cells, along with the downregulation of Mlxipl mRNA (Fig. 3E, F). Functionally, Mbnl1 overexpression protected against PA-impaired cell viability and proliferation in Min6 cells (Fig. 3G, H), as well as PA-induced apoptosis of Min6 cells (Fig. 3I). Overexpression of Mbnl1 also rescued PA-suppressed insulin secretion in Min6 cells (Fig. 3J). PA-reduced circMlxipl and Mbnl1, as well as PA-induced Mlxipl mRNA and ChREBP, were reversed by Mbnl1 overexpression (Fig. 3K, L). These findings suggest that Mbnl1 is identfied as a RBP of pre-Mlxipl, and it is implicated in circMlxipl-mediated protection in lipotoxic β-cells.

### Hdac3 is implicated in circMlxipl-mediated protection in lipotoxic β-cells

**Fig. 4:**
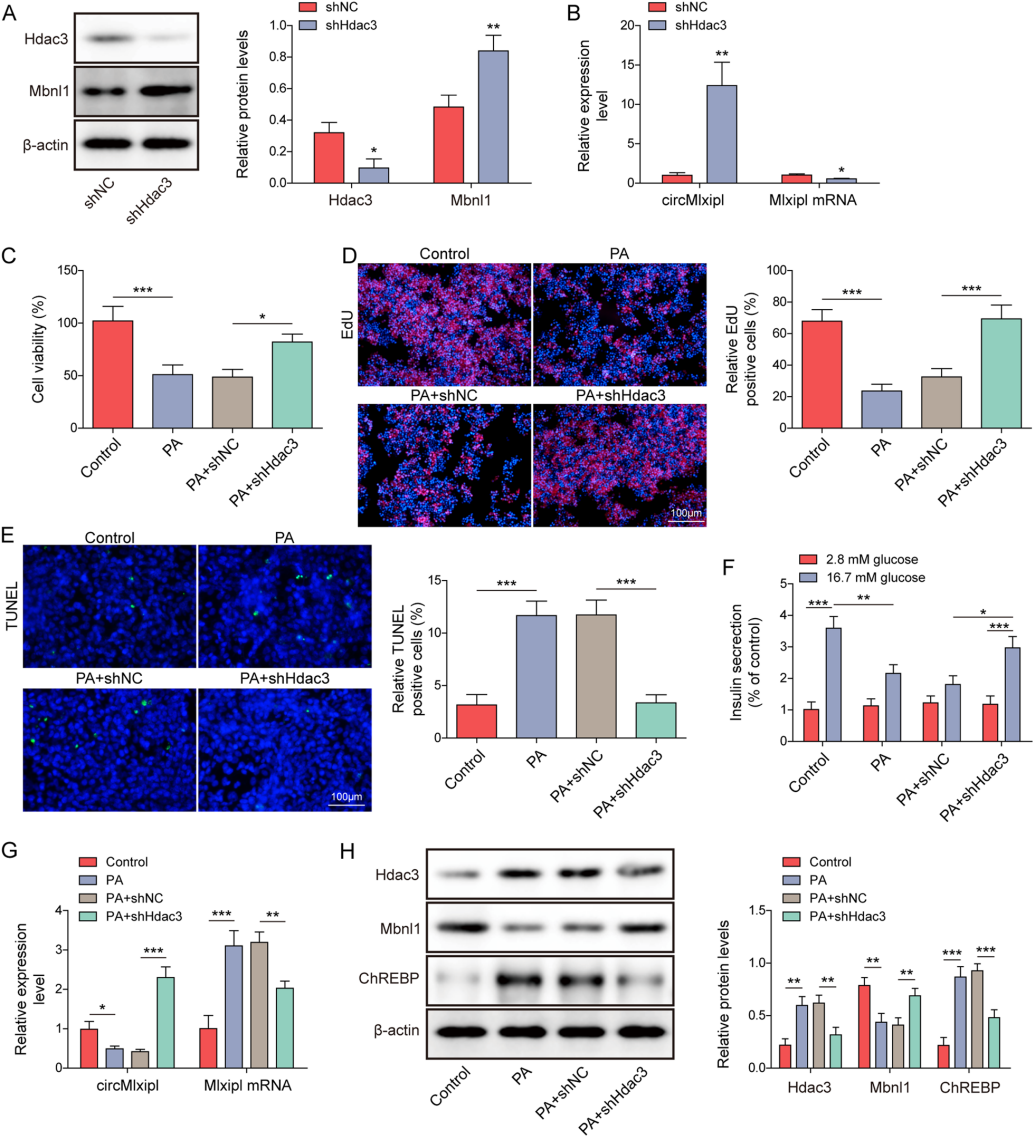
Hdac3 is implicated in circMlxipl-mediated protection in lipotoxic β-cells. Min6 cells were transfected with shHdac3 or shNC, then the expression levels of circMlxipl, Mlxipl mRNA, Hdac3 and Mbnl1 were evaluated. **A** The protein levels of Hdac3 and Mbnl1 were detected by western blot. **B** The levels of circMlxipl and Mlxipl mRNA in Min6 cells were detected by qRT-PCR. Min6 cells were transfected with shHdac3 or shNC, then treated with palmitic acid (PA), cell viability, proliferation, cell apoptosis and insulin secretion were performed. **C** Cell viability of Min6 cells was monitored by MTT assay. **D** Cell proliferation was detected by EdU incorporation assay with quantitative analysis. Scale bar, 100 μm. **E** Cell apoptosis was assessed by TUNEL assay with quantitative analysis. Scale bar, 100 μm. **F** Insulin secretion was detected by GSIS. Min6 cells were transfected with shHdac3 or shNC, then treated with palmitic acid (PA), the expression levels of circMlxipl, Mlxipl mRNA, Hdac3, Mbnl1 and ChREBP were evaluated. **G** The levels of circMlxipl and Mlxipl mRNA in Min6 cells were detected by qRT-PCR. **H** The protein levels of Hdac3, Mbnl1 and ChREBP were detected by western blot. *P < 0.05; **P < 0.01; ***P < 0.001

To test if Hdac3 was involved in circMlxipl-mediated protection in lipotoxic β-cells, knockdown studies were carried out. As expected, silencing of Hdac3 decreased Hdac3 protein expression and Mlxipl mRNA level, but upregulated the expression of Mbnl1 and circMlxipl in Min6 cells (Fig. 4A, B). Similarly, PA-suppressed viability and proliferation of Min6 cells were rescued by Hdac3 knockdown (Fig. 4C, D). Silencing of Hdac3 also counteracted PA-triggered apoptosis of Min6 cells, and led to a rebound of insulin secretion (Fig. 4E, F). Moreover, knockdown of Hdac3 reversed PA-mediated changes of circMlxipl, Mlxipl mRNA, Mnbl1 and ChREBP in Min6 cells (Fig. 4G, H). Together, these findings suggest that Hdac3 is implicated in circMlxipl-mediated protection in lipotoxic β-cells.

### Hdac3 serves as a transcriptional repressor of Mbnl1

**Fig. 5:**
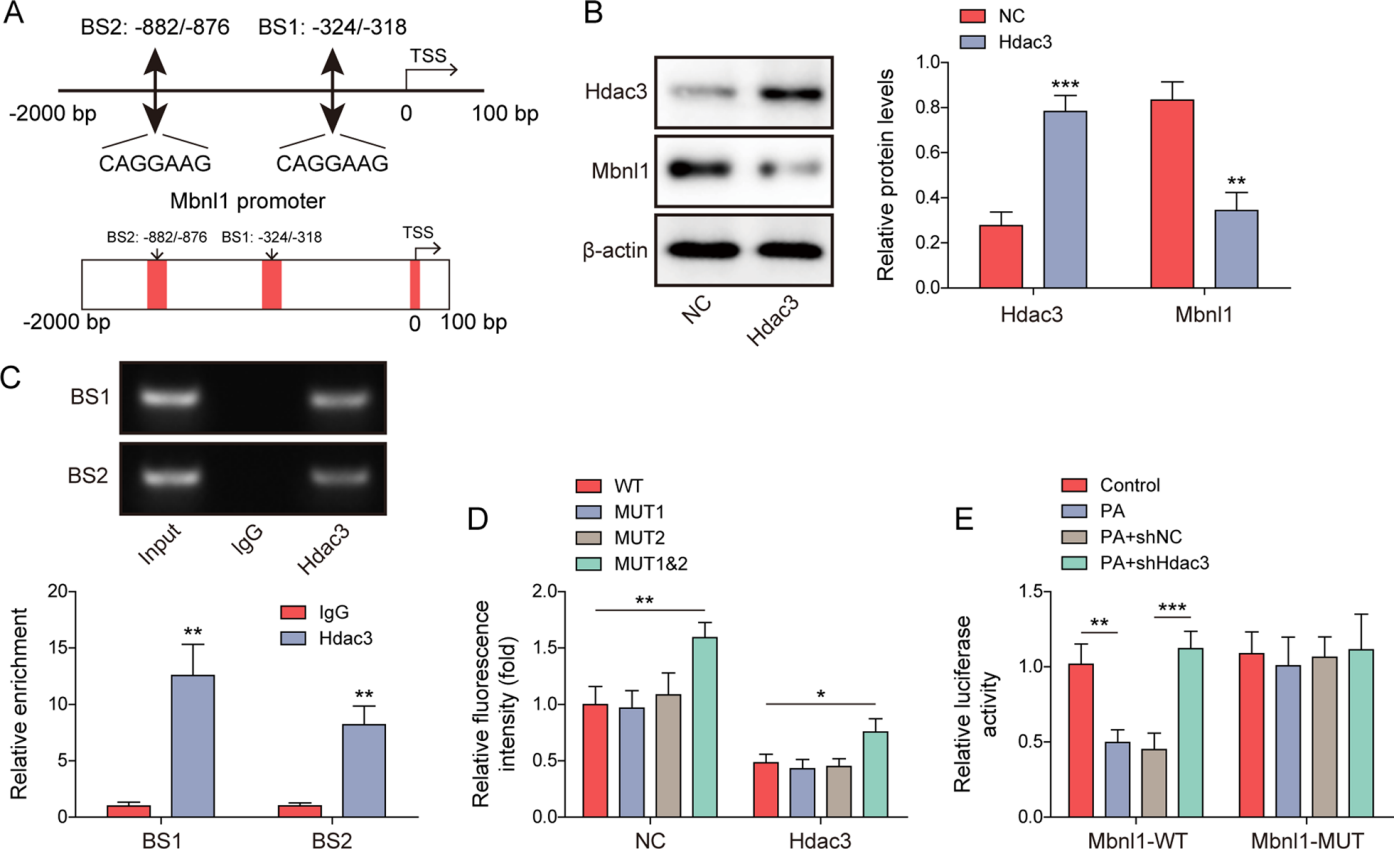
Hdac3 serves as a transcriptional repressor of Mbnl1. **A** The putative binding sites between Mbnl1 promoter and Hdac3 were predicted by AnimalTFBD. **B** The protein levels of Hdac3 and Mbnl1 were detected by western blot. **C** The associations between Mbnl1 BS1 domain and Hdac3, as well as Mbnl1 BS2 domain and Hdac3, were detected by ChIP assay. Normal IgG served as a negative control. **D**, **E** The relative luciferase activity of transfected Min6 cells were assessed by dual- luciferase reporter assay. *P < 0.05; **P < 0.01; ***P < 0.001

AnimalTFDB predicted two putative binding sites on the promoter region of Mbnl1, including BS1 p(-324/-318) and BS2 p(-882/-876) (Fig. 5A). Western blot showed that Hdac3 overexpression increased Hdac3 expression, but decreased Mbnl1 protein level in Min6 cells (Fig. 5B). Antibody against Hdac3 successfully immunoprecipitated both BS1 and BS2 fragments in Min6 cells as detected by ChIP assay (Fig. 5C). Luciferase assay revealed that Hdac3 overexpression reduced the luciferase activity of Mbnl1, compared with corresponding control. Double mutation of BS1 & BS2 resulted in an induction of luciferase activity, and similar trend was also found in Hdac3-overexpressing Min6 cells (Fig. 5D). Single mutation of either BS1 or BS2 had no effect on the luciferase activity, suggesting that both BS1 and BS2 domains were required for the binding between Hdac3 and Mbnl1 promoter. In addition, PA downregulated the luciferase activity of wild-type Mbnl1 (Mbnl1-WT), whereas this negative effect was rescued by Hdac3 knockdown. No remarkable change of luciferase activity was observed in Mbnl1 mutant (Mbnl1-MUT)-overexpressing Min6 cells (Fig. 5E). These data indicate that Hdac3 acts as a transcriptional repressor of Mbnl1, and BS1/BS2 binding domains are required for the interaction between Hdac3 and Mbnl1 promoter.

### Hdac3/Mbnl1 suppresses the formation of circMlxipl to enhance ChREBP expression in lipotoxic β-cells

**Fig. 6:**
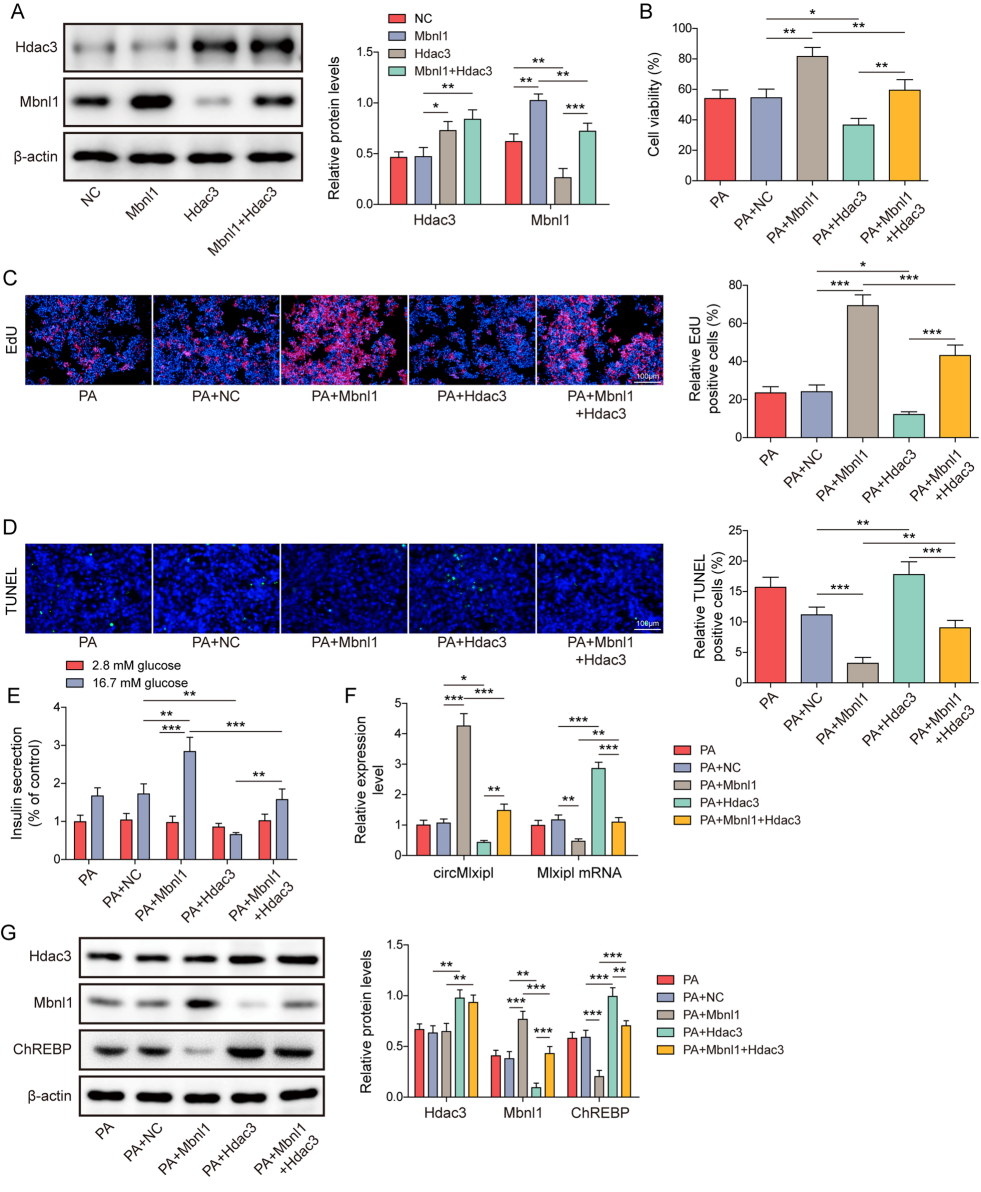
Hdac3/Mbnl1 suppresses the formation of circMlxipl to enhance ChREBP expression in lipotoxic β-cells. **A** The protein levels of Hdac3 and Mbnl1 in treated Min6 cells were detected by western blot. **B** Cell viability of Min6 cells was monitored by MTT assay. **C** Cell proliferation was detected by EdU incorporation assay with quantitative analysis. Scale bar, 100 μm. **D** Cell apoptosis was assessed by TUNEL assay with quantitative analysis. Scale bar, 100 μm. **E** Insulin secretion was detected by GSIS. **F** The levels of circMlxipl and Mlxipl mRNA in Min6 cells were detected by qRT-PCR. **G** The protein levels of Hdac3, Mbnl1 and ChREBP in treated Min6 cells were detected by western blot. *P < 0.05; **P < 0.01; ***P < 0.001

To test the hypothesis, overexpression and functional experiments were next conducted in Min6 cells. As presented in Fig. 6A, Hdac3 overexpression successfully increased Hdac3 expression, and downregulated Mbnl1. Additionally, overexpression of Mbnl1 reversed Hdac3-downregulated Mbnl1 in Min6 cells (Fig. 6A). Consistently, Mbnl1 overexpression exhibited protective effects on cell viability, proliferation, apoptosis and insulin secretion in lipotoxic β-cells (Fig. 6B–E). On the contrary, overexpression of Hdac3 potentiated PA-impaired cell viability, proliferation, apoptosis and insulin secretion in Min6 cells, while co-transfection of Hdac3 and Mbnl1 reversed Hdac3-exacerbated impairments (Fig. 6B–E). Furthermore, Mbnl1 overexpression induced circMlxipl and Mbnl1 levels, but decreased Mlxipl mRNA and ChREBP level in lipotoxic β-cells. Hdac3 overexpression exerted opposite effects, whereas Hdac3-mediated changes of these molecules were counteracted by Mbnl1 overexpression (Fig. 6F, G). These findings indicate that Hdac3 inhibits the formation of circMlxipl in an Mbnl1-dependent manner, thus inducing ChREBP expression to exacerbate lipotoxicity in Min6 cells.

### Knockdown of circMlxipl inhibits Rbbp6-mediated ubiquitin-proteasomal degradation of ChREBP in lipotoxic β-cells

**Fig. 7:**
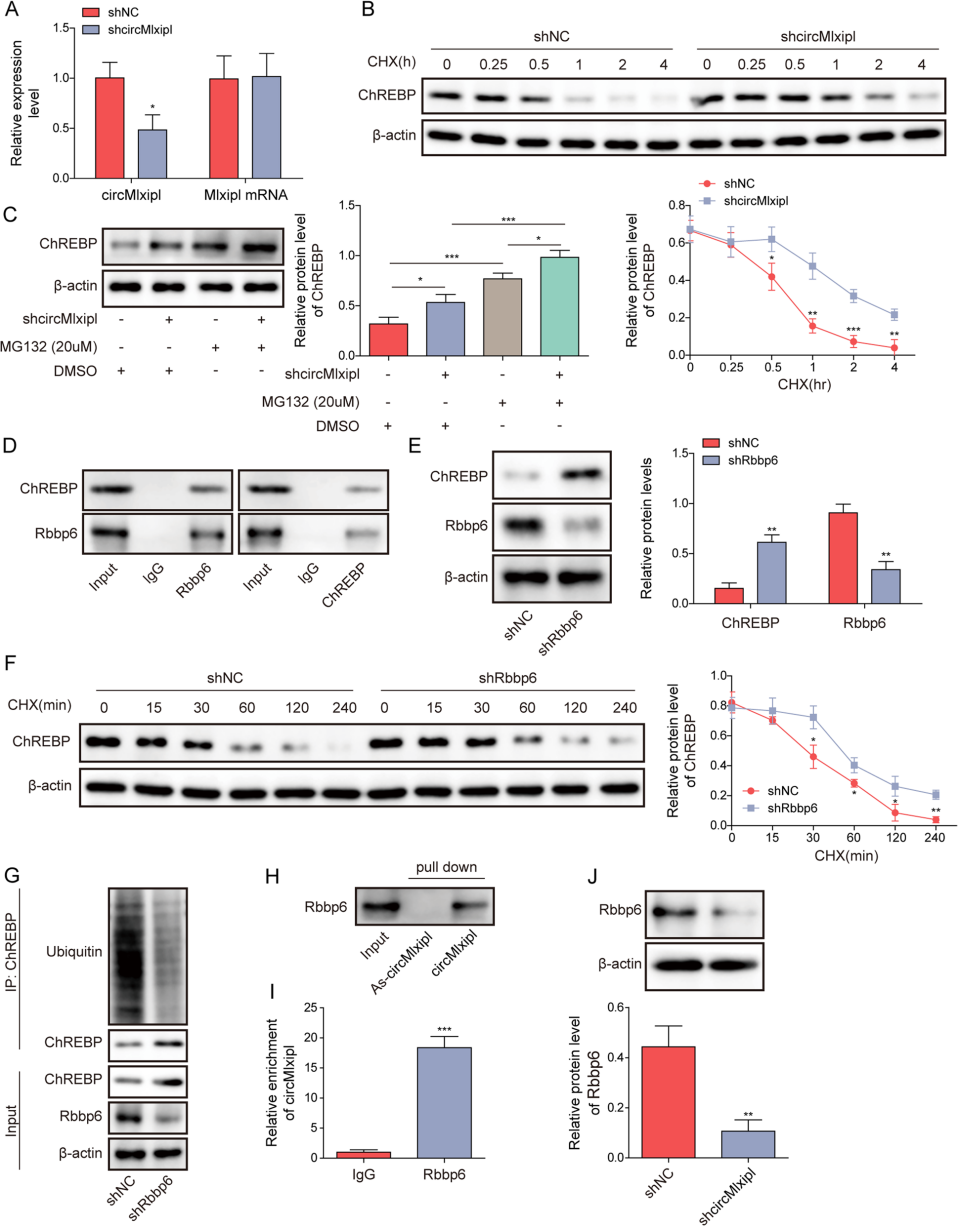
Knockdown of circMlxipl inhibits Rbbp6-mediated ubiquitin-proteasomal degradation of ChREBP in lipotoxic β-cells. **A** The levels of circMlxipl and Mlxipl mRNA in control or circMlxipl-knockdown Min6 cells were detected by qRT-PCR. **B** The protein stability of ChREBP in CHX-treated control, circMlxipl-knockdown Min6 cells was detected by western blot with quantitative analysis. **C** Min6 cells were transfected with sh-NC or sh-circMlxipl and treated with MG132. The protein level of ChREBP was detected by western blot. **D** The direct interaction between ChREBP and Rbbp6 was detected by co-IP. Normal IgG or whole cell lysates was used as a negative or input control, respectively. **E** Min6 cells were transfected with sh-NC or sh-Rbbp6. The protein levels of ChREBP and Rbbp6 were detected by western blot. **F** The protein stability of ChREBP in CHX-treated control, Rbbp6-knockdown Min6 cells was detected by western blot with quantitative analysis. **G** The ubiquitination of ChREBP was examined by co-IP. **H**, **I** The association between circMlxipl and Rbbp6 was detected by RNA pull-down and RIP assays. Anti-sense probe and normal IgG served as negative controls for RNA pull-down assay and RIP, respectively. **J** Min6 cells were transfected with sh-NC or sh-circMlxipl. The protein level of Rbbp6 was detected by western blot. **K** The direct interaction between ChREBP and Rbbp6 was detected by co-IP. Normal IgG or whole cell lysates was used as a negative or input control, respectively. **L**, **M** Min6 cells were transfected with sh-NC/sh-circMlxipl or/and Rbbp6 overexpression construct, followed by vehicle or PA treatment. The protein levels of ChREBP and Rbbp6 were detected by western blot. *P < 0.05; **P < 0.01; ***P < 0.001

We next sought to investigate the mechanism underlying circMlxipl-mediated regulation of ChREBP. Ubibrowser predicted that E3 ubiquitin ligase Rbbp6 possibly mediated the ubiquitin-proteasomal degradation of ChREBP, and RPISeq predicted the potential interaction between Rbbp6 and circMlxipl. Therefore, knockdown studies were performed to test if circMlxipl/Rbbp6 mediated the turnover of ChREBP in lipotoxic β-cells. As shown in Fig. 7A, transfection of sh-circMlxipl reduced circMlxipl level, while silencing of circMlxipl had no effect on Mlxipl mRNA level in Min6 cells (Fig. 7A). In addition, lack of circMlxipl slowed down the degradation of ChREBP in the presence of CHX (Fig. 7B), and proteasome inhibitor MG132 led to a further rebound of ChREBP in Min6 cells (Fig. 7C). These findings suggest that circMlxipl is involved in the ubiquitin-proteasomal degradation of ChREBP. Co-IP revealed that anti-Rbbp6 antibody successfully enriched ChREBP in Min6 cells, vice versa (Fig. 7D). As anticipated, silencing of Rbbp6 upregulated ChREBP expression in Min6 cells (Fig. 7E), and the degradation of ChREBP was inhibited in Rbbp6-knockdown Min6 cells in the presence of CHX (Fig. 7F). Consistent with these findings, decreased ubiquitination of ChREBP was also observed in Rbbp6-knockdown Min6 cells (Fig. 7G), indicating that Rbbp6 is required for ChREBP degradation in Min6 cells. Moreover, RNA pull-down and RIP assays unequivocally revealed that there was a direct association between circMlxipl and Rbbp6 in Min6 cells (Fig. 7H, I). Silencing of circMlxipl showed the decreased Rbbp6 expression (Fig. 7J). Loss of circMlxipl also suppressed the binding between Rbbp6 and ChREBP as detected by co-IP (Fig. 7K). Furthermore, circMlxipl knockdown increased ChREBP expression, but decreased the protein level of Rbbp6. Rbbp6 overexpression induced Rbbp6 protein level, along with the reduction of ChREBP, and overexpression of Rbbp6 counteracted sh-circMlxipl-decreased Rbbp6 and increased ChREBP expression in Min6 cell in the absence or presence of PA (Fig. 7L, M). These data suggest that circMlxipl knockdown inhibites Rbbp6-mediated ubiquitin-proteasomal degradation of ChREBP in lipotoxic β-cells.

### Hdac3 knockdown or Mbnl1 overexpression alleviates diabetes symptoms through circMlxipl-regulated ChREBP in vivo

**Fig. 8:**
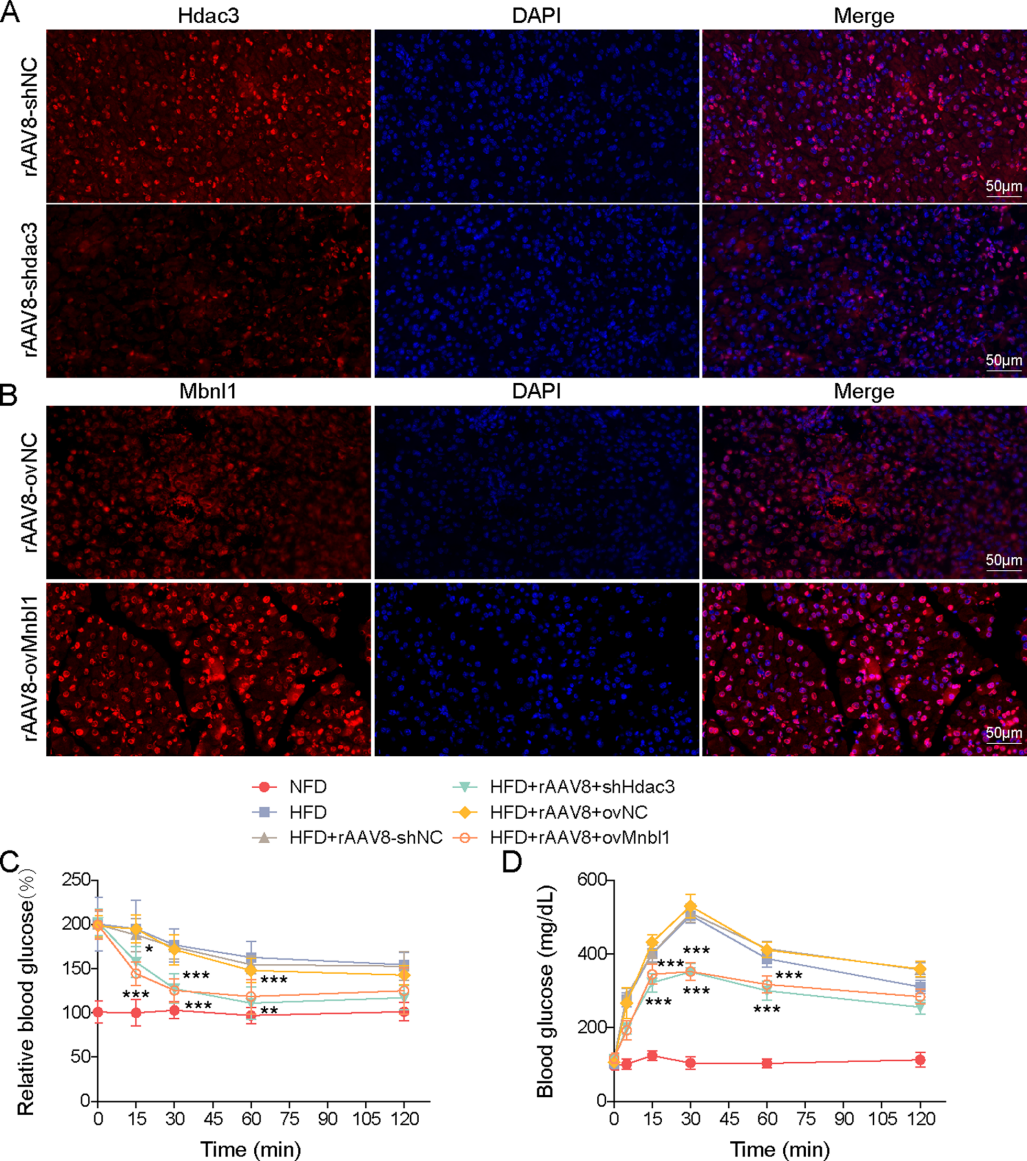

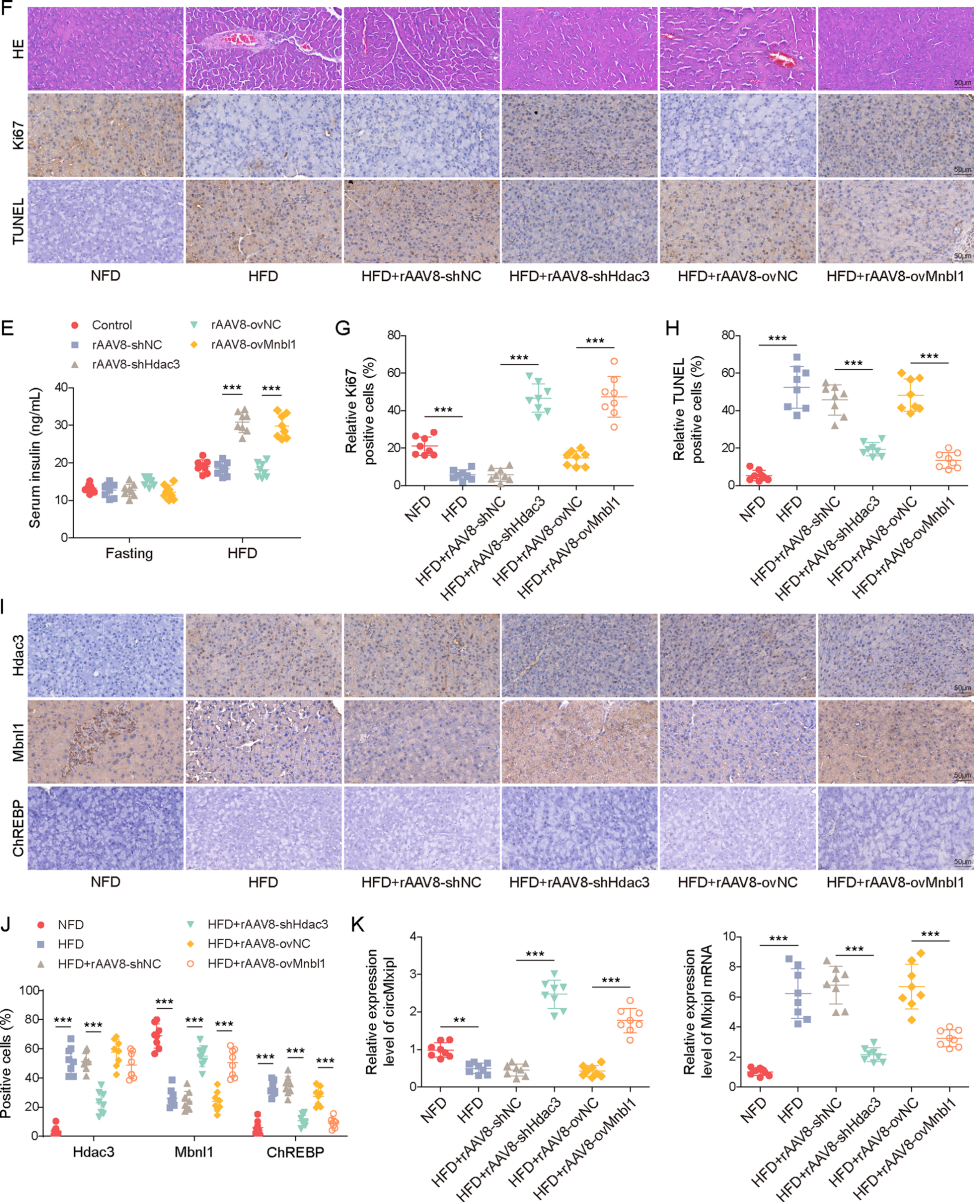
Hdac3 knockdown or Mbnl1 overexpression alleviated diabetes symptoms through circMlxipl-regulated ChREBP in vivo. **A** The expression level of Hdac3 in the pancreas was detected by IF staining. Scale bar, 50 μm. **B** The expression level of Mbnl1 in the pancreas was detected by IF staining. Scale bar, 50 μm. ITTs (**C**), and GTTs (**D**) were performed in HFD-fed mice. **E** The serum level of insulin was measured by ELISA assay. **F** The histological changes of mouse pancreas were monitored by H&E staining. Scale bar, 50 μm. **G** The immunoactivity of Ki-67 in the pancreas was detected by IHC staining with quantitative analysis. Scale bar, 50 μm. **H** Cell apoptosis in the pancreas was assessed by TUNEL assay with quantitative analysis. Scale bar, 50 μm. **I** The levels of circMlxipl and Mlxipl mRNA in the pancreas were detected by qRT-PCR. **J** The immunoactivities of Hdac3, Mbnl1 and ChREBP in the pancreas were detected by IHC staining. Scale bar, 50 μm. *P < 0.05; **P < 0.01; ***P < 0.001

To validate the findings in vivo, functional studies were conducted in diet-induced T2DM C57BL/6 J mouse model. AAV8-mediated Hdac3 knockdown or Mbnl1 overexpression were performed in HFD mice (Fig. 8A, B). Moreover, Hdac3 knockdown or Mbnl1 overexpression significantly improved HFD-induced insulin resistance and glucose intolerance (Fig. 8C, D). ITTs showed that the glucose levels of mice with high Mbnl1 expression were decreased within 15 to 60 min, in comparison with that in control group (Fig. 8C). As shown in Fig. 8E, loss of Hdac3 or Mnbl1 overexpression had no remarkable effect on the serum insulin level in the fasting condition, while Hdac3 silencing or Mnbl1 overexpression dramatically increased serum insulin level in HFD-fed mice. H&E staining showed that the pancreas derived from NFD mice exhibited normal morphology and the residual islets of HFD mice showed shrunk and disrupted structures (Fig. 8F). IHC analysis and TUNEL assay also showed decreased Ki67-positvie cells and increased TUNEL-positive cells in the pancreas of HFD mice (Fig. 8G, H). By contrast, in vivo Hdac3 knockdown or Mbnl1 overexpression protected against HFD-induced morphological impairments, accompanied with rescued proliferation and apoptosis (Fig. 8F–H). In addition, IHC analysis and qRT-PCR revealed that HFD decreased Mbnl1 and circMlxipl expression in the pancreas, but increased Hdac3, ChREBP and Mlxipl mRNA levels in vivo. Silencing of Hdac3 counteracted these changes, and overexpression of Mbnl1 reversed HFD-induced changes of these molecules, except for Hdac3 (Fig. 8I, J). Collectively, these findings indicate that Hdac3 knockdown or Mbnl1 overexpression alleviated diabetes symptoms, possibly through circMlxipl-regulated ChREBP in vivo.

## Discussion

Over the past few decades, the prevalence and cases of diabetes have been increasing due to several risk factors, such as aging, dietary excess, obesity and sedentary lifestyle (Yu et al. 2024). Pancreatic β-cells, the most abundant islet cell type, are the only insulin-secreting cells (Jain et al. 2022). In T2DM, a decrease of β-cell mass and increase of β-cell apoptosis have been observed which is accompanied with the reduction of insulin secretion (Dludla et al. 2023). A number of studies have illustrated that lipotoxicity is one of the mechanisms underlying β-cell failure (Galicia-Garcia et al. 2020; Vilas-Boas et al. 2021). In this study, we demonstrated that Hdac3/Mbnl1 was implicated in the regulation of circMlxipl back-splicing in lipotoxic β-cells, thus decreasing ChREBP expression in an Rbbp6-dependent manner to inhibit lipotoxicity-induced β-cell failure. These data shed light on the targeted therapy for diabetes.

Numerous circRNAs have been identified in islets by RNA sequencing. Among 346 dysregulated circRNAs in diabetic mice, circMlxipl is the second remarkably decreased circRNA (Wu et al. 2020). Consistent with the bioinformatics analysis, we reported that circMlxipl was downregulated in db/db and HFD mice, as well as in PA-treated Min6 cells. In line with previous reports, Mlxipl mRNA and ChREBP were upregulated in these in vivo and in vitro models (Jing et al. 2016; Katz et al. 2022; Herman et al. 2012). ChREBP has been identified in adipose tissue and β-cells (Herman et al. 2012; Zhang et al. 2015). ChREBP contributes to glucose-stimulated proliferation of β-cells (Zhang et al. 2015), and chronic overexpression of ChREBP results in loss of β-cell mass, β-cell apoptosis and diabetes (Katz et al. 2022), suggesting the critical role of ChREBP in the maintenance of β-cell mass. The functional experiment also illustrated that circMlxipl exerted its protective effects via decreasing ChREBP expression in lipotoxic β-cells, suggesting that ChREBP serves as a downstream effector of circMlxipl in β-cells.

Emerging evidence supports the role of RBPs in diabetes and diabetes complications (Nutter and Kuyumcu-Martinez 2018; Zhang et al. 2022). In this study, Mbnl1 was identified as an RBP of pre-Mlxipl. Mbnl1 is a splicing factor that facilitates the inclusion of insulin receptor exon 11 (Sen et al. 2010). In addition, more recent studies have also reported that Mbnl1 ameliorates diabetic nephropathy via miR-130a-3p/STAT3 axis in response to Metformin (Jiang et al. 2020). In lipotoxic β-cells, we also observed the protective role of Mbnl1, and increased expression of Mbnl1 induced circMlxipl level. Alternative splicing is involved in circRNA circularization and dysregulation of circRNA (Zhang et al. 2016). However, the mechanism by which Mbnl1 regulates circMlxipl back-splicing still merits further investigation in the future study. Moreover, Hdac3 transcriptionally regulated Mbnl1 expression in β-cells. Previous study has demonstrated that Hdac3-specific inhibitor MS-275 protects β-cells against cytokine-induced apoptosis in type 1 diabetes (Chou et al. 2012), as well as from palmitate-induced cell death in diabetes (Plaisance et al. 2014). Mechanistic studies have reported that inhibition of Hdac3 is implicated in the regulation of gluconeogenesis, oxidative metabolism and hepatic FGF21 expression in diabetes (Meier and Wagner 2014). Besides the known protective functions of Hdac3 in β-cell apoptosis (Chou et al. 2012; Meier and Wagner 2014; Plaisance et al. 2014), we reported a novel mechanism by which Hdac3 preserves β-cells against lipotoxicity.

E3 ubiquitin ligase specifically recognizes its target substrate and transfer ubiquitin from E2 enzyme to the target protein (Yang et al. 2021). Rbbp6 is a RING finger-domain E3 ubiquitin ligase which is upregulated in various cancers and associated with poor prognosis (Li et al. 2007; Chibi et al. 2008; Moela et al. 2014; Motadi et al. 2011). For instance, Rbbp6 acts as a negative regulator of the tumor suppressor p53 by facilitating Hdm2-p53 interaction and mediating ubiquitin-proteasomal degradation of p53 (Li et al. 2007). In this study, we reported that loss of circMlxipl or Rbbp6 slowed down the degradation of ChREBP in the presence of CHX, indicating that circMlxipl or Rbbp6 regulates ChREBP at the post-translational level. Proteasome inhibitor MG132 blocked the turnover of ChREBP, suggesting the key role of ubiquitin–proteasome pathway in circMlxipl-mediated regulation of ChREBP. Additionally, ChREBP was identified as a novel substrate of Rbbp6 in β-cells. circRNAs could mediate protein stability (Jiaxin et al. 2021). Consistent with these findings, silencing of circMlxipl decreased Rbbp6 expression, and reduced ChREBP degradation in β-cells. Previous study has demonstrated that ChREBP constitutively expresses in the nucleus due to its potent transactivity (Herman et al. 2012). It is speculated that circMlxipl and Rbbp6 might form an RNA–protein complex in the cytosol, while knockdown of circMlxipl may reduce Rbbp6 from this complex and inhibit the ubiquitin-proteasomal degradation of ChREBP.

In conclusion, we found that Hdac3 transcriptionally regulated the expression of splicing factor Mbnl1, thus modulating back-splicing of circMlxipl. In lipotoxic β-cells, circMlxipl overexpression reduced ChREBP expression through Rbbp6-mediated ubiquitin-proteasomal degradation, thereby inhibiting β-cell apoptosis. These findings illustrated the protective role of circMlxipl in lipotoxicity-induced β-cell failure and its potential therapeutic value.

## Data Availability

No datasets were generated or analysed during the current study.

## Abbreviations

AAV: Adeno-associated viral
ChIP: Chromatin immunoprecipitation
ChREBP: Carbohydrate-response element-binding protein-β
CHX: Cycloheximide
circRNA: Circular RNAs
co-IP: Co-immunoprecipitation
EdU: 5-Ethynyl-2’-deoxyuridine
FISH: Fluorescence in situ hybridization
gDNA: Genomic DNA
GSIS: Glucose-induced insulin secretion
Hdac3: Histone deacetylase 3
H&E: Hematoxylin and Eosin
HFD: High fat diet
IHC: Immunohistochemistry
INSR: Insulin receptor
KRH: Krebs–Ringer HEPES
Mbnl1: Muscleblind-like 1
Mlxipl: MLX Interacting Protein Like
MUT: Mutant
NEFA: Non-esterified fatty acid
NFD: Normal control diet
PA: Palmitic acid
PFA: Paraformaldehyde
Rbbp6: Retinoblastoma binding protein 6
RBP: RNA binding protein
RIP: RNA immunoprecipitation
T2DM: Type 2 diabetes mellitus
TUNEL: Terminal deoxynucleotidyl transferase dUTP nick end labeling
vg: Viral genome
WT: Wild type

## References

[CR1] Chibi M, et al. RBBP6 interacts with multifunctional protein YB-1 through its RING finger domain, leading to ubiquitination and proteosomal degradation of YB-1. J Mol Biol. 2008;384:908–16. 18851979 10.1016/j.jmb.2008.09.060

[CR2] Chou DH, et al. Inhibition of histone deacetylase 3 protects beta cells from cytokine-induced apoptosis. Chem Biol. 2012;19:669–73. 22726680 10.1016/j.chembiol.2012.05.010 PMC3383610

[CR3] Corbin KL, et al. A practical guide to rodent islet isolation and assessment revisited. Biol Proced Online. 2021;23:7. 33641671 10.1186/s12575-021-00143-x PMC7919091

[CR4] Diabetes Canada Clinical Practice Guidelines Expert C, et al. Pharmacologic glycemic management of type 2 diabetes in adults: 2020 update. Can J Diabetes. 2020;44:575–91. 32972640 10.1016/j.jcjd.2020.08.001

[CR5] Dludla PV, et al. Pancreatic beta-cell dysfunction in type 2 diabetes: Implications of inflammation and oxidative stress. World J Diabetes. 2023;14:130–46. 37035220 10.4239/wjd.v14.i3.130 PMC10075035

[CR6] Galicia-Garcia U, et al. Pathophysiology of type 2 diabetes mellitus. Int J Mol Sci. 2020;21:6275. 32872570 10.3390/ijms21176275 PMC7503727

[CR7] Gupta A, et al. Role of UPP pathway in amelioration of diabetes-associated complications. Environ Sci Pollut Res Int. 2021;28:19601–14. 33660172 10.1007/s11356-021-12781-5

[CR8] Herman MA, et al. A novel ChREBP isoform in adipose tissue regulates systemic glucose metabolism. Nature. 2012;484:333–8. 22466288 10.1038/nature10986 PMC3341994

[CR9] Huang A, Zheng H, Wu Z, Chen M, Huang Y. Circular RNA-protein interactions: functions, mechanisms, and identification. Theranostics. 2020;10:3503–17. 32206104 10.7150/thno.42174 PMC7069073

[CR10] Huang S, et al. Histone deacetylase 3 inhibition alleviates type 2 diabetes mellitus-induced endothelial dysfunction via Nrf2. Cell Commun Signal. 2021;19:35. 33736642 10.1186/s12964-020-00681-z PMC7977318

[CR11] Jain C, Ansarullah BS, Lickert H. Targeting pancreatic beta cells for diabetes treatment. Nat Metab. 2022;4:1097–108. 36131204 10.1038/s42255-022-00618-5

[CR12] Jiang X, et al. Metformin reduces the senescence of renal tubular epithelial cells in diabetic nephropathy via the MBNL1/miR-130a-3p/STAT3 pathway. Oxid Med Cell Longev. 2020;2020:8708236. 32104542 10.1155/2020/8708236 PMC7035567

[CR13] Jiaxin C, et al. Circular RNA circRHOBTB3 represses metastasis by regulating the HuR-mediated mRNA stability of PTBP1 in colorectal cancer. Theranostics. 2021;11:7507–26. 34158864 10.7150/thno.59546 PMC8210600

[CR14] Jing G, Chen J, Xu G, Shalev A. Islet ChREBP-beta is increased in diabetes and controls ChREBP-alpha and glucose-induced gene expression via a negative feedback loop. Mol Metab. 2016;5:1208–15. 27900263 10.1016/j.molmet.2016.09.010 PMC5123192

[CR15] Katz LS, et al. Maladaptive positive feedback production of ChREBPbeta underlies glucotoxic beta-cell failure. Nat Commun. 2022;13:4423. 35908073 10.1038/s41467-022-32162-x PMC9339008

[CR16] Li L, et al. PACT is a negative regulator of p53 and essential for cell growth and embryonic development. Proc Natl Acad Sci U S A. 2007;104:7951–6. 17470788 10.1073/pnas.0701916104 PMC1876553

[CR17] Malakar P, et al. Insulin receptor alternative splicing is regulated by insulin signaling and modulates beta cell survival. Sci Rep. 2016;6:31222. 27526875 10.1038/srep31222 PMC4985653

[CR18] Meier BC, Wagner BK. Inhibition of HDAC3 as a strategy for developing novel diabetes therapeutics. Epigenomics. 2014;6:209–14. 24811789 10.2217/epi.14.11

[CR19] Moela P, Choene MM, Motadi LR. Silencing RBBP6 (Retinoblastoma Binding Protein 6) sensitises breast cancer cells MCF7 to staurosporine and camptothecin-induced cell death. Immunobiology. 2014;219:593–601. 24703106 10.1016/j.imbio.2014.03.002

[CR20] Motadi LR, Bhoola KD, Dlamini Z. Expression and function of retinoblastoma binding protein 6 (RBBP6) in human lung cancer. Immunobiology. 2011;216:1065–73. 21676486 10.1016/j.imbio.2011.05.004

[CR21] Nauck MA, Wefers J, Meier JJ. Treatment of type 2 diabetes: challenges, hopes, and anticipated successes. Lancet Diabetes Endocrinol. 2021;9:525–44. 34181914 10.1016/S2213-8587(21)00113-3

[CR22] Nutter CA, Kuyumcu-Martinez MN. Emerging roles of RNA-binding proteins in diabetes and their therapeutic potential in diabetic complications. Wiley Interdiscip Rev RNA. 2018;9:29280295. 10.1002/wrna.1459 PMC5815912 29280295

[CR23] Plaisance V, et al. The class I histone deacetylase inhibitor MS-275 prevents pancreatic beta cell death induced by palmitate. J Diabetes Res. 2014;2014:195739. 25610877 10.1155/2014/195739 PMC4294305

[CR24] Robson-Doucette CA, et al. Beta-cell uncoupling protein 2 regulates reactive oxygen species production, which influences both insulin and glucagon secretion. Diabetes. 2011;60:2710–9. 21984579 10.2337/db11-0132 PMC3198081

[CR25] Schleicher E, et al. Definition, classification and diagnosis of diabetes mellitus. Exp Clin Endocrinol Diabetes. 2022;130:S1–8. 35451038 10.1055/a-1624-2897

[CR26] Sen S, et al. Muscleblind-like 1 (Mbnl1) promotes insulin receptor exon 11 inclusion via binding to a downstream evolutionarily conserved intronic enhancer. J Biol Chem. 2010;285:25426–37. 20519504 10.1074/jbc.M109.095224 PMC2919106

[CR27] Vilas-Boas EA, Almeida DC, Roma LP, Ortis F, Carpinelli AR. Lipotoxicity and beta-cell failure in type 2 diabetes: oxidative stress linked to NADPH oxidase and ER stress. Cells. 2021;10:3328. 34943836 10.3390/cells10123328 PMC8699655

[CR28] Wu L, et al. Circ-Tulp4 promotes beta-cell adaptation to lipotoxicity by regulating soat1 expression. J Mol Endocrinol. 2020;65:149–61. 33064661 10.1530/JME-20-0079 PMC7576671

[CR29] Yang Q, Zhao J, Chen D, Wang Y. E3 ubiquitin ligases: styles, structures and functions. Mol Biomed. 2021;2:23. 35006464 10.1186/s43556-021-00043-2 PMC8607428

[CR30] Yu G, et al. Lessons and applications of omics research in diabetes epidemiology. Curr Diab Rep. 2024;24:27–44. 38294727 10.1007/s11892-024-01533-7 PMC10874344

[CR31] Zhang P, et al. Induction of the ChREBPbeta isoform is essential for glucose-stimulated beta-cell proliferation. Diabetes. 2015;64:4158–70. 26384380 10.2337/db15-0239 PMC4657577

[CR32] Zhang XO, et al. Diverse alternative back-splicing and alternative splicing landscape of circular RNAs. Genome Res. 2016;26:1277–87. 27365365 10.1101/gr.202895.115 PMC5052039

[CR33] Zhang S, et al. Post-transcriptional control by RNA-binding proteins in diabetes and its related complications. Front Physiol. 2022;13:953880. 36277184 10.3389/fphys.2022.953880 PMC9582753

